# Five years follow-up of schoolchildren infected with schistosomiasis in Niger: evidence of the benefit of a regular praziquantel administration on the reinfection

**DOI:** 10.1186/1753-6561-5-S1-P57

**Published:** 2011-01-10

**Authors:** Zilahatou Tohon, Halima Boubacar, Ramatou Yahaya, Biba Abdou, Pascal Boisier

**Affiliations:** 1 Unité d’Epidémiologie, Centre de Recherche Médicale et Sanitaire, BP 10887 Niamey, Niger; 2 Laboratoire d'Epidémiologie, Centre Pasteur du Cameroun, BP 1274, Yaoundé, Cameroun

## Background

The WHO’s objective regarding schistosomiasis control is to maintain a low burden by maintaining lower parasitic charges in endemic regions. The schistosomiasis and helminthiasis control program was launched in 2004 in Niger. Although yearly praziquantel treatment does not clear entirely schistosomiasis worms in people, it allows reducing parasitic loads and thus avoids serious renal complications. A study was carried out to estimate the annual prevalence of *Schistosoma haematobium* infection and its related morbidity among schoolchildren living in 5 endemic villages between 2004 and 2009.

## Results

A longitudinal follow-up of school age children was undertaken with pre-treatment examination and follow-up each year prior to mass drug administration campaign. Prevalence of schistosomiasis and anaemia was assessed through interview, urine examination, ultrasound of the kidney and urinary tract, and measurement of haemoglobin. At baseline, *S. haematobium* infection prevalence was 76.8% among the 1017 enrolled schoolchildren. Among the infected, 30.2% excreted more than 50 eggs/10 ml of urine. After 4 and 5 years of follow-up, urinary schistosomiasis was respectively retrieved among 44.3% and 26.5% of the 680 and 581 children, respectively. A proportion of 23.0% of uninfected schoolchildren at baseline became infected at year 4. Conversely, 48% of schoolchildren with positive egg count at baseline were found negative at year 4 (p<0.001). These results remained significant taking into account the village. At baseline, anaemia was present in 63.1% of children, and its prevalence significantly decreases with increasing age. This prevalence was reduced to 51.8%, 40.5% and 25.9% at years 2, 3 and 5 of follow-up respectively.

## Conclusion

The prevalence of ultrasound bladder abnormalities was 37.7% at baseline and this prevalence dropped to 2.6% at year 5 of follow-up. Regular praziquantel treatment had positive impact on the prevalence of schistosomiasis as well as on its intensity and on the reinfection rate. These results encourage proceeding with the national schistosomiasis and geohelminthiasis control programme.

